# Population health management guiding principles to stimulate collaboration and improve pharmaceutical care

**DOI:** 10.1108/JHOM-06-2017-0146

**Published:** 2018-04-09

**Authors:** Betty Steenkamer, Caroline Baan, Kim Putters, Hans van Oers, Hanneke Drewes

**Affiliations:** 1 Tranzo, Tilburg School of Social and Behavioral Sciences, Tilburg University, Tilberg, The Netherlands; 2 Center for Nutrition, Prevention and Health Services, National Institute for Public Health and the Environment, Bilthoven, The Netherlands; 3 Department of Health Care Governance, Institute for Health Policy and Management, Erasmus University Rotterdam, Rotterdam, The Netherlands; 4 The Netherlands Institute for Social Research, The Hague, The Netherlands

**Keywords:** Health care, Governance, Qualitative research, Strategy, Knowledge sharing, Pharmaceuticals

## Abstract

**Purpose:**

A range of strategies to improve pharmaceutical care has been implemented by population health management (PHM) initiatives. However, which strategies generate the desired outcomes is largely unknown. The purpose of this paper is to identify guiding principles underlying collaborative strategies to improve pharmaceutical care and the contextual factors and mechanisms through which these principles operate.

**Design/methodology/approach:**

The evaluation was informed by a realist methodology examining the links between PHM strategies, their outcomes and the contexts and mechanisms by which these strategies operate. Guiding principles were identified by grouping context-specific strategies with specific outcomes.

**Findings:**

In total, ten guiding principles were identified: create agreement and commitment based on a long-term vision; foster cooperation and representation at the board level; use layered governance structures; create awareness at all levels; enable interpersonal links at all levels; create learning environments; organize shared responsibility; adjust financial strategies to market contexts; organize mutual gains; and align regional agreements with national policies and regulations. Contextual factors such as shared savings influenced the effectiveness of the guiding principles. Mechanisms by which these guiding principles operate were, for instance, fostering trust and creating a shared sense of the problem.

**Practical implications:**

The guiding principles highlight how collaboration can be stimulated to improve pharmaceutical care while taking into account local constraints and possibilities. The interdependency of these principles necessitates effectuating them together in order to realize the best possible improvements and outcomes.

**Originality/value:**

This is the first study using a realist approach to understand the guiding principles underlying collaboration to improve pharmaceutical care.

## Background

In order to provide high quality care while keeping health care systems affordable and accessible, many countries are moving toward a population-based approach. The assumption underlying a population-based approach is that to achieve better population health and quality of care and a reduction in cost growth (Triple Aim (TA); Berwick *et al.*, 2008), collaboration is needed within and across public health, health care, social care and community services (Alderwick *et al.*, 2015). In Western countries, initiatives, often referred to as population health management (PHM) initiatives, have emerged to close the gap between health and community services (Steenkamer *et al.*, 2017). For example, in the USA, accountable care communities and private sector accountable care organizations work together in multisector initiatives designed to improve population health in communities and neighborhoods (Alley *et al.*, 2016). PHM initiatives have also emerged in the Netherlands. In 2013, the Dutch Ministry of Health, Welfare and Sport designated several of these initiatives as pioneer sites (Drewes *et al.*, 2016). The pioneer sites are monitored and evaluated by the Dutch Monitor of Pioneer sites Population Management of the Dutch National Institute for Public Health and the Environment (Struijs *et al.*, 2015). These initiatives, serving over 2 million people, represent partnerships between health care insurance companies, primary care groups, hospitals, municipalities and community-based organizations, including regional patient organizations. All initiatives aim to improve health and provide better care at lower costs for the regional population by bridging clinical and community services (Drewes *et al.*, 2016). The pioneer sites started in 2013 with both care-related and pre-conditional interventions such as the organization of a governance structure for, for example, the development of a shared agenda and coordination of activities and communication.

Improving pharmaceutical care was one of the first subjects addressed within the monitored Dutch PHM initiatives. In recent years, health care insurance companies had tried different policies, such as outcome-based funding for effective prescription of medicine by general practitioners, in an attempt to improve the quality of pharmaceutical care and to control the cost of medicines. However, the expenditure on pharmaceutical care barely dropped (Batenburg *et al.*, 2015). Moreover, considerable regional variation remained such as regional variation in prescription rates of expensive drugs and regional variation in adherence to medical guidelines (Lambooij *et al.*, 2016). Possible explanations for these differences were amongst others the lack of transmural agreements between hospitals and primary care organizations (Kerpershoek *et al.*, 2012). Such an agreement provides, for example, clarity about roles and responsibilities between general practitioners, medical specialists and pharmacists with regard to the pharmaceutical management of patients and contributes to the exchange of pharmaceutical expertise (Kerpershoek *et al.*, 2012; Snyder *et al.*, 2010). Also doctors were not always aware of the cost of medicine or neither took costs into account in their decision making with regard to what drugs to prescribe, nor were doctors always aware of what drugs were covered in what formulary (Kerpershoek *et al.*, 2012). The PHM initiatives assumed that a coordinated intervention including multidisciplinary collaboration aiming to promote safe use and efficient prescription of medicine both by general practitioners and, in hospitals (extramural medication), would be better able to achieve financial savings and improve quality of care. Therefore, the Dutch PHM initiatives introduced a range of strategies to stimulate collaboration between medical specialists, general practitioners, (hospital and community) pharmacists and regional patient organizations such as joint development of a pharmaceutical formulary. Besides, in their overall aim to bridge prevention, care and welfare in the upcoming years, PHM initiatives expected to build upon the new structures and arrangements (e.g. new work groups for the development of new data technologies or shared shavings contracts) that were developed and tested in this intervention for the first time.

In recent years, several strategies to improve collaboration on pharmaceutical care have already been pointed out in the literature such as organizing face-to-face discussions between doctors and pharmacists (Joseph *et al.*, 2017; Patel, 2016; Chui *et al.*, 2014). However, these strategies do not always work out as expected (Snyder *et al.*, 2010; Joseph *et al.*, 2017; Chui *et al.*, 2014). Research has shown that depending on the circumstances in which strategies are implemented, and the motivations of people and organizations to act upon the opportunities and resources that strategies offer in these circumstances, the same strategy can have different results (Pawson, 2013). In general, studies have insufficiently taken into account both contextual and motivational factors that contribute to the explanation of how and why collaborative strategies achieved their results (Saul *et al.*, 2013; Glasgow *et al.*, 2012; Greenhalgh *et al.*, 2004). With regard to pharmaceutical care, no studies could be found describing both the circumstances in which collaborative strategies were implemented and the motivational factors that influence the outcomes of these strategies. Up till now, just one study (Chui *et al.*, 2014) analyzed the motivations of physicians and pharmacists with regard the developing and sustaining collaboration. However, this study did not include contextual factors that influence motivations of people and organizations. Taking into account the complexity of the various strategies, contextual factors and motivations of different professionals and organizations, as revealed in the collaborative adaptive health network (CAHN) framework (Steenkamer *et al.*, 2018), is important to understand which strategies work in which situations and how and why these strategies contribute to organizing collaboration to improve pharmaceutical care. However, up till now, an insight into the relationships between strategies, contextual factors, mechanisms and outcomes of strategies with regard to pharmaceutical care is lacking.

Therefore, the aim of this study is to identify how and why specific strategies stimulate collaboration in order to improve pharmaceutical care. More specifically, this study will identify the guiding principles that highlight how collaboration can be stimulated to improve pharmaceutical care while taking into account local constraints and possibilities.

## Methods

This evaluation was informed by the realist evaluation method of Willis *et al.* (2016). Traditional realist evaluations aim to provide an understanding of “what works, for whom, in what context, to what extent and how and why it works to produce outcomes?” (Pawson, 2013). As such, the underlying principles of a realist approach are the links between respectively: strategies (*S*), contexts (*C*), mechanisms (*M*) and outcomes (*O*). The main focus of traditional realist evaluations is on generating or testing theories by focusing on how particular contexts (*C*) trigger changes in the reasoning and behavior of human actors (mechanisms (*M*); Wong *et al.*, 2016). Instead of providing a theory, those working in complex systems such as the PHM initiatives benefit most from guiding principles based on theory-driven, contextually relevant strategies (*S*) that are associated with specific outcomes (*O*) (Saul *et al.*, 2013; Willis *et al.*, 2016). Therefore, in contrast to traditional realist reviews, the focus of this study was to point out the guiding principles and the contexts and mechanisms by which these principles operate. Strategies are considered a directed course of action that produce (desired) proximal, intermediate or final (process) outcomes (McKeown, 2011). In our study, strategies are related to collaboration to improve pharmaceutical care. Contexts are the different sociocultural, relational, economic, political and historical configuration such as financial incentives or the history of the working relationship (Glasgow *et al.*, 2012). Mechanisms are the changes in the behavior or reasoning of stakeholders triggered by changes in contexts like a growing sense of urgency of PHM initiatives to improve pharmaceutical care.

This realist evaluation included several iterative stages: engagement of experts and reference panels; data collection; and analyzing, synthesizing and interpretation of data. As per realist evaluations’ methodology and in line with the RAMESES standards (Wong *et al.*, 2013), an expert and reference panel were engaged to ensure the evaluation was grounded in the needs of the knowledge user and was consistent with current international expertise and knowledge. The research team consisted of experts with national and international experience and expertise in health system transformation and PHM. As a reference panel, the existing Advisory Committee of the Dutch Monitor of Pioneer sites Population Management was involved, including scientists and representatives of the Ministry of Health, Welfare and Sports and of the PHM initiatives. In addition, a local reference panel was involved, consisting of representatives of all seven PHM initiatives included in this review. In a kick-off meeting, the research question, methodology and the CAHN framework (Steenkamer *et al.*, 2018) to be used for the analysis and synthesis of the data in this study were presented to the experts and the local reference panel. The framework is based on an international inventory of the literature on collaborative efforts cross-linking public health, health care and social and community services to achieve the TA. The framework describes eight components (relations, social forces, accountability, leadership, resources, finance, regulations and market). Most components consist of three or more subcomponents. The (sub)components contain the available theories and insights into the relationships between strategies, contextual factors, mechanisms and outcomes of collaborative efforts cross-linking public health, health care, social care and community services. The experts and local references agreed to the research question, methodology and framework used in this study.

Between January 2016 and June 2016, data were collected during focus groups and individual interviews of seven PHM initiatives in total. Focus groups were held in 4 PHM initiatives with a total of 26 participants (general practitioners, pharmacists, medical specialists (internal medicine, and cardiology), representatives of health care insurers and patient organizations, and project and program managers). For three PHM initiatives, individual interviews were held with a program manager or pharmacist (three interviews in total) (an overview of the participants and information about the structure and organization of the PHM initiatives and the pharmaceutical interventions is available on request).

To become familiar with the intervention improving pharmaceutical care and the PHM region, the authors collected additional data such as pharmaceutical toolkits describing the agreements and procedures regarding the new multidisciplinary approach to pharmaceutical care, which were developed by the PHM initiatives. Also during the kick-off meeting, participants were asked to share their experiences with the new strategies PHM initiatives practiced.

A semi-structured interview guide was used to support the interview process (available on request). At two points during the interview, all interviewees were asked to write down their lessons regarding which strategies worked or failed in improving collaboration on pharmaceutical care. The first time was at the start of the interview enabling the discussion about these lessons during the focus group or during the individual interview. The second time was after the presentation of the eight components of the CAHN framework, enabling interviewees to add lessons that came to mind upon viewing the eight components. All lessons were discussed, and additional information regarding strategies, contextual factors, underlying mechanisms and outcomes were retrieved during the interviews. The interviews were audio recorded and transcribed verbatim.

In realist evaluations, analysis, synthesis and interpretation of the data tend to occur alongside each other during the evaluation process (Pawson, 2006). Data analysis of the data collected during the interviews was done using Microsoft Excel® and the MAXQDA software. Using MAXQDA, texts containing information with regard to links between context-specific strategies, contextual factors, mechanisms and outcomes related to the context specific strategies were given a codename reflecting the essence of the passage. Strategies were identified by descriptions of courses of action at the board or operational level. Outcomes of strategies were identified by descriptions of unintended or intended proximal, intermediate or final process results of collaborative strategies to improve pharmaceutical care. Contexts in which the strategy was implemented were recognized by descriptions of circumstantial factors on a local, regional or national level. Mechanisms were recognized by descriptions of changes in the reasoning or the way stakeholders acted upon the opportunities and resources the strategies offered in the specific context. The quality of the relations between the elements of each link was guided by the requirements of a realist review (Wong *et al.*, 2013). The codes were arranged in MAXQDA along the according (sub)components of the CAHN framework. Next, the overview in MAXQDA of the links between strategies, contexts, mechanisms and outcomes per (sub)component was converted into a Microsoft Excel® spreadsheet. Furthermore, the name from the PHM initiative from which each link originated was added to every link. This spreadsheet was used to identify the guiding principles. Per (sub)component, context-specific strategies related to specific outcomes that were put forward by at least two PHM initiatives were grouped into guiding principles. The research team shared the evidence gathered for each guiding principle on a regular basis. As a result, the team formed an evolving understanding of the contextual factors enabling or constraining the likelihood of the guiding principles to be effective and the mechanisms by which the specific outcomes of the guiding principles were reached.

The research team and reference panels reflected and commented upon the whole research process. In addition, a researcher outside the research team (LB) verified the analysis, synthesis and interpretation process. Furthermore, results were presented and discussed during a feedback meeting with representatives of four of the participating PHM initiatives and one PHM initiative that did not participate in this study. Finally, based on this feedback, the guiding principles and accompanying contextual factors and mechanisms were debated and refined within the research team.

### Ethics approval

Ethics approval from the Psychological Ethical Review Committee at Tilburg University (EC-2015.54) was received in October 2015.

## Results

A total of ten guiding principles enhancing collaboration to improve pharmaceutical care were identified. The strategies as well as the contexts and mechanisms by which these guiding principles operate are shown in Table I. Some strategies, contexts and mechanisms appear across different guiding principles. This illustrates how particular strategies influence collaboration to improve pharmaceutical care in multiple ways. The next section elaborates on the guiding principles. First, per guiding principles, groups of strategies related to specific outcomes will be described. Next, we give an account of these strategies, the contextual factors enabling or constraining the effectiveness of these strategies and the mechanisms by which outcomes are reached.

### Guiding principles, strategies, contextual factors and underlying mechanisms

#### Guiding principle 1: create agreement and commitment based on a long-term vision

PHM initiatives implemented two different strategies to organize agreement on and commitment to a coordinated intervention including multidisciplinary collaboration regarding pharmaceutical care (see Table I). First, all PHM initiatives invited a number of regional stakeholders within the care sector to engage in improving pharmaceutical care. Predating the PHM initiatives, too often interventions were undertaken by single organizations and professionals (hospitals, general practitioners) with limited benefits to the target population, let alone lower pharmaceutical costs. This contextual factor induced a sense of urgency among regional stakeholders to organize a coalition of the willing to improve pharmaceutical care in line with the TA. Another contextual factor was dissatisfaction among stakeholders with health care insurance companies purchasing care products for the lowest negotiated price on a yearly basis. This purchasing process elicited competition among providers in the regional market, which was already characterized by a history of mono-disciplinary interventions. This induced readiness for change in stakeholders for a regional multidisciplinary approach toward pharmaceutical care based on a long-term vision, as is reflected in the following quote:

[…] Many single projects [aiming to improve pharmacy care] in the past have failed, and now we aim for sustainable regional collaboration based on health, quality and costs. So there is a vision behind it which is the driving force behind everything we are going to do in the future

(I2, health care insurer representative).

Second, five PHM initiatives facilitated stakeholders to jointly develop a business plan for substituting brand for generic medicines including a new financial incentive model (e.g. shared-savings contract). A contextual factor enabling this strategy was a growing sense of urgency mainly from the health care insurance companies to counter ongoing high prescriptions of expensive drugs with little effect on health improvement. For the providers, this growing sense of urgency was also fueled by the fact that failing to cooperate would have negative financial consequences (see also guiding principle 7). This contextual factor induced feelings of problem ownership and encouraged stakeholders to reach agreement and commitment regarding lowering of prescription rates of expensive drugs and changes in ways of working, capacity and finance. However, in four PHM initiatives, other contextual factors such as corporate restructuring of stakeholders’ organizations hindered the process of agenda setting and the timing of engagement of individual organizations. Consideration of strategic, financial and substantive arguments at the board level of participating organizations took more time. In these organizations, prioritizing between what they regarded as a small-scale project and their own restructuring process delayed the process of gaining agreement and commitment to multidisciplinary collaboration on pharmaceutical care.

#### Guiding principle 2: foster cooperation and representation at board level

PHM initiatives fostered cooperation and representation at steering committee level to encourage leaders of regional care groups, hospitals and pharmacies to invest in multidisciplinary collaboration on pharmaceutical care. This study identified two strategies. The first strategy was that PHM initiatives sought opportunities to stimulate multidisciplinary collaboration (five PHM initiatives). Within the context of expected positive revenues gained through the introduction of shared savings contracts, this strategy enabled leaders to cooperate for three reasons. First, leaders felt obliged to prove that a multidisciplinary approach to pharmaceutical care could be successful. Given the historically disappointing results of a mono-disciplinary approach to improve pharmaceutical care, multidisciplinary agreement on lowering the prescription rates of expensive drugs and enhancement of drug safety guaranteed these quick wins. Second, leaders reasoned that these quick wins could give them the opportunity to create a financial buffer to support future projects within the PHM initiatives. Lastly, they presumed that cooperation would increase the visibility of PHM initiatives on a regional and national level.

The second strategy was installing the right people at the right time in the right place (all PHM initiatives). PHM initiatives differed in the way they organized representation of pharmacists depending on which knowledge (content or strategic) was needed during the process. Pharmacists were represented in the steering committee of the PHM initiative (four PHM initiatives), or regional legal entity (one PHM initiative). In addition, all PHM initiatives organized representation of pharmacists at the project level, and some had an additional steering group for this specific intervention:

[…] at an early stage, you often need representatives that are supported by their organizations and have the capability to think strategically. After 3 or 4 meetings you also need knowledge […] this issue is insufficiently recognized […]

(I3, pharmacist).

Representation of pharmacists at the level of the steering committee of PHM initiatives increased the possibility to channel and discuss problems and to adjust policy regarding pharmaceutical care. Consequently, the involvement of pharmacists in other projects of the PHM initiatives increased. However, pharmacists felt less involved when only represented on a lower level. On the other hand, representation in a project group led to pharmacists investing more time and in-depth knowledge in the project in comparison to participation in the steering committee. The regional legal entity combined the best of both worlds: generating more involvement and visibility of pharmacists in regional projects and more investment of time and expertise.

#### Guiding principle 3: use the layered governance structure (steering committee and operational level)

All PHM initiatives used a layered governance structure for escalation and facilitation purposes to direct and enable multidisciplinary collaboration. The governance structure of most PHM initiatives comprises a steering committee, one or more working groups and sometimes an executive or management committee. Two strategies were identified. First, all PHM initiatives made conscious use of information within the layered governance structure for escalation or facilitation purposes. This strategy was profitable within the context of solving problems for which additional background information or exchange of information at a certain level within the governance structure was needed. Managers, for instance, learned more about the professional, organizational or regional sensitivities and interests during steering committee meetings. Consciously using information for escalation and facilitation purposes helped to generate commitment to modify prescription behavior and adjust ways of working in line with the agreed upon pharmaceutical protocol.

Second, all PHM initiatives made conscious use of the skills and influencing power of PHM managers and professional experts for escalation or facilitation purposes within the layered governance structure. In situations where professionals had different paces of adjusting to the new multidisciplinary working processes, this strategy worked positively for those colleagues who had worked alongside each other for years. By having PHM managers confront professionals on their pace of adjustment to the new ways of working, instead of having colleagues confronting each other, it was ensured that professional relationships were not put under pressure. In contexts in which stakeholders had different interests and levels of commitment within the layered governance structure, the same strategy generated confidence mechanisms among stakeholders. Stakeholders were of the opinion that PHM managers and professional experts such as medical specialists who, at the same time, hold a professorship were more capable of highlighting the differences and similarities in interests and commitment due to their so-called independent position within the layered governance structure. They were assumed to know the different organizational cultures and to have good communication and persuasion skills. The contribution of PHM managers and experts’ ability to enhance confidence levels was an important precondition to direct PHM initiatives toward multidisciplinary collaboration and thus for launching the pharmacy intervention (six PHM initiatives).

#### Guiding principle 4: create awareness at all levels

Six PHM initiatives applied three strategies to ensure awareness at all levels about the need to improve pharmaceutical care, thus allowing the development of new pharmaceutical protocols and working processes. First, PHM initiatives organized continuous interaction and communication between health care insurance companies, regional care providers and patient organizations. The managers played an important role in the execution of this strategy by serving as a link between parties and by organizing discussions (see also guiding principles 3-7). A contextual factor enabling this strategy was the development of a pharmacy toolkit, which enabled discussions regarding guidelines and scientific knowledge, and health care insurance claims data. These discussions generated several mechanisms. First of all, they enabled the re-examination of existing pharmacotherapies with the intention of reducing costs without losing the quality of care. Furthermore, these discussions generated taking into account multiple medical guidelines (general practitioners and various medical specialists), which, although evidence-based, sometimes contradicted each other:

For example with regard to the lipid level, there is a difference between general practitioners and cardiologists with respect to the cut-off values that should be pursued

(I7, cardiologist).

Discussing differences made stakeholders more aware of each other’s point of view. Despite these differences, consensus regarding new pharmaceutical protocols was reached. Lastly, the discussions also stimulated the re-examination of existing working processes and professional roles in order to modify them in line with the new multidisciplinary pharmacy protocol (five PHM initiatives). For example, agreements were reached about the time frame within which patients had to be informed, the number of substitution consultations that should be executed and who was responsible for drafting the pharmaceutical advices and for the substitution consultations:

[…] differences become transparent and negotiable during discussion. Sometimes we reached an agreement, sometimes not. Most of the time consensus was reached. Also negative emotions became apparent. In the past, differences were often not understood, people felt that others did not cooperate but did not know why

(I15, project manager).

A second strategy was making use of existing consultation situations. An enabling contextual factor of this strategy was the pre-existing quality of consultations between medical specialists, general practitioners, pharmacists, physician assistants and patients (three PHM initiatives). Joining existing consultation situations in which people already knew and trusted each other created a safe situation for discussing medication policies and ways of working. Consequently, creating awareness for needed changes was easier than in situations where trust levels were low (three PHM initiatives).

A third strategy in the same context generated similar mechanisms. PHM initiatives developed information leaflets and made information available in waiting rooms to support communication with patients of the new medication policy. Discussing this information in a trusted environment increased awareness of patients that a generic drug would have the same, or even better results with lower costs, and reduced feelings of mistrust toward the new drug efficacy (five PHM initiatives).

#### Guiding principle 5: enable interpersonal links at all levels

PHM initiatives organized interpersonal links to establish openness to (new) knowledge, ideas and a more multidisciplinary perspective regarding pharmaceutical care to support changes in norms, values and ways of working. This study recognized three strategies and one enabling contextual factor influencing these three strategies. This contextual factor was the already increased collaboration within primary care and between primary and secondary care. For instance, for years most general practitioners and pharmacists were engaged in pharmacotherapy consultation groups and knew each other on a personal level. Two strategies were identified. The first strategy of PHM initiatives was joining these already existing consultations (five PHM initiatives) (see also guiding principle 4) and second was organizing regional multidisciplinary meetings to share best practices or the pharmacy protocols that were developed (four PHM initiatives).

The third strategy PHM initiatives undertook was to include a range of different professionals in these regional meetings. These strategies enhanced trust, recognition and acknowledgment of each other’s contribution and scientific knowledge (two PHM initiatives). In three PHM initiatives, continuous sharing of experiences in the region led to installment of a regional multidisciplinary pharmaceutical committee (see also guiding principle 2).

#### Guiding principle 6: create a learning environment

PHM initiatives created a learning environment that supported the use of measurements within a feedback loop (plan-do-check-act). This study identified two strategies. First, PHM initiatives organized data input and/or new tools to match the information needs of professionals (all PHM initiatives). Until then, health care insurance claims data provided insufficient insight into the degree of improvement of prescription practices due to, for example, a lack of a historical overview and insufficient insight for general practitioners into repeat prescriptions of medical specialists (all PHM initiatives). Also the time frame within which changes in prescriptions were reflected in the data and the level of feedback was insufficient (five PHM initiatives). These contextual factors enabled the strategy to enhance motivation of professionals to engage in the feedback loop.

Second, to support data input and tool development, PHM initiatives organized capacity and knowledge of data technology, data analysis and synthesizing data into meaningful information. Information was seen as important in creating awareness of inefficient pharmaceutical care. Insufficient capacity and knowledge put pressure on professionals and health care insurance companies to establish either internal or external capacity and knowledge to support data input and tool development. The former meant either health care insurance companies (four PHM initiatives) or professionals themselves (one PHM initiative) took the lead in organizing capacity and knowledge. Professional control led to an immediate enhancement of the level and frequency of data feedback. Two PHM initiatives organized external capacity and knowledge. In one of these PHM initiatives, this resulted in the development of a business-intelligence tool that links selected data from pharmacy, primary care and laboratory registries in order to track and decrease the use of high-cost, low-quality services for specific diseases.

#### Guiding principle 7: organize shared responsibility

PHM organized shared responsibility for multidisciplinary improvement of regional pharmacy care. This study recognized two strategies. First, five PHM initiatives organized a new incentive design for multidisciplinary responsibility to improve pharmaceutical care. Contextual factors enabling this strategy were the fact that the old incentive design (e.g. separate incentives for general practitioners and pharmacists) did not fit the new multidisciplinary agreement of PHM initiatives. Also, leaders felt an urgent need to improve pharmaceutical care, as they were not pleased with negative financial incentives for not following the formulary (also see guiding principle 1). In addition, leaders expected shared savings to encourage multidisciplinary responsibility by letting stakeholders share realized savings that were associated with certain results (e.g. X% brand converted into generic within certain time frame). However, a constraining contextual factor of the strategy was the difference in basic principles of professionals and organizations that became apparent during the development of the pharmacy protocol, such as differences in cut-off values and scores or setting of the benchmark (also see guiding principles 4 and 6). For instance, teaching hospitals only wanted to be benchmarked with other teaching hospitals, as their patient population is very different from community hospitals. PHM initiatives took these differences into account while designing these new incentive designs.

The second strategy was to organize an adherence design, based on social forces like peer reviewing in which general practitioners, pharmacists and medical specialists discussed each other’s results. Because stakeholders were dissatisfied with pre-existing individual and regional prescription rates of expensive drugs, peer reviewing induced a shift to multidisciplinary accountability resulting in a higher participation rate of providers than mono-disciplinary accountability (five PHM initiatives). Also, peer reviewing in a multidisciplinary context prompted strong feelings of motivation for achieving better performances, especially if stakeholders already had positive experiences with the plan-do-check-act cycle (five PHM initiatives).

#### Guiding principle 8: adjust financial strategies to the market context

PHM initiatives implemented two financial strategies to strive for the best pharmacy therapy for the lowest price, given the market context. First, two PHM initiatives took into account market factors and trends of pharmaceutical products such as prices, applicability of products and expiration dates of patents to define the pharmacy formulary. On the basis of this formulary, health care insurance companies negotiated with manufacturers on the basis of efficiency and quality:

If you look at the price of Spiriva for COPD patients, sixty percent of the low budget medication is Spiriva […]. But the patent of Spiriva expires this year […] Now the costs are 80 or 90 euros and this can drop to a couple of euros […]

(I12, pharmacist).

The insurance companies successfully challenged manufacturers to lower pricing rates per serving in order for them to be included into the formulary. Although the formulary gave financial and qualitative advantages, it was also criticized for reducing the freedom of choice of medicine:

For asthma/COPD products, we looked per interchangeable groups of medicines on the basis of qualitative criteria. How medicine should be applied played an important role in this choice. […] And now the insurer is asking the pharmaceutical market to react on this formulary. The manufacturers don’t know the formulary content, but were asked to provide the best possible price. If manufacturers are lower in price than the ones on the formulary, and the quality of the medicine does not differ much, then such a manufacturer can still end up on the formulary. In this way we try to combine quality with efficiency

(I15, program manager).

Second, two PHM initiatives organized financial insight to support treatment options, starting with people with multimorbidity. Within the context of striving for the best pharmaceutical therapy for the lowest price, this strategy induced deliberate use of financial outcomes and combining this data with clinical data and patient preferences:

For CVRM you can make estimates how much risk you want to take. […] You cannot change everything at once, so you would like to discuss with patients ‘if you lose five kilos the chance of getting a heart attack will be reduced with 10%, and if you stop smoking then it will drop with another 30%, and when you take your medicine the chances will reduce even further with another 5%. So what is feasible for you? By bringing together data with regard to clinical outcomes, patient preferences and financial data e.g. option 1 would be the cheapest solution and option 3 would lead to a financial break even

(I29, program manager).

As a result, PHM initiatives were capable of weighing individual treatment options as well as improving health care purchasing processes in the regional market (two PHM initiatives). Although combining data raised political concern related to privacy laws and regulations, one PHM initiative derived legitimacy for the experiment based on support expressed by a visiting political delegate.

#### Guiding principle 9: organize mutual gains

PHM initiatives organized mutual gains to develop a financial buffer and put the initiative “on the map.”

This study identified two strategies. The first strategy of PHM initiatives was to focus on “low-hanging fruit” to gain quick wins. Because leaders expected positive revenues gained through the introduction of shared savings contracts, several distribution scenarios of potential savings were under debate at the start of the intervention. One of these scenarios was directing all savings back to the professionals executing the intervention (see also guiding principles 2 and 7). However, discussions in society at large about unequal distribution of bonuses influenced the debate. Three PHM initiatives were motivated to funnel net savings back to serve as a financial buffer for future interventions that would benefit the health of the regional community.

The second strategy of PHM initiatives was to enhance the visibility of the PHM initiatives (see also guiding principle 2). Improving pharmaceutical care was just the first intervention of a growing regional PHM program as initiatives wanted to address medical and social determinants of health of the regional community. The pressure of PHM initiatives to work toward a comprehensive regional program led to a focus on achieving financial wins (four PHM initiatives). These quick wins not only symbolized successful regional multidisciplinary collaboration, but also the potential of PHM initiatives to continue their journey and enhance their regional and national visibility.

#### Guiding principle 10: align regional agreements with national policies and regulations

PHM initiatives implemented two strategies. First, PHM initiatives indicated at the earliest possible moment when existing regulations did not match new regional agreements. Four PHM initiatives stated that a constraining contextual factor for this strategy was the “preference policy” of health care insurance companies. Although health care insurance companies agreed to the regional pharmaceutical formulary of PHM initiatives, in hindsight, one medication group did not match the national preference policy of insurance companies. This contextual factor led to risk factors at the level of the treatment relationship, as PHM initiatives assumed all medicines within the formulary would be reimbursed. Providers sometimes had to deal with dissatisfaction of patients due to the miscommunication about reimbursement of medicine. Furthermore, PHM initiatives risked damaging the accountability relationship between the insurance company and the initiatives. Although, eventually, the national preference policy was adjusted to the regional policy regarding the aforementioned medication group, providers were only reimbursed from this moment onwards. Other constraining contextual factors were the existing walls regarding funding systems between sectors and disciplines:

Although we have made agreements to provide integrated care, we face the current fragmented funding streams. So, integrated agreements were unraveled into separate pieces in order for them to be separately funded in line with the existing system. This is not easy, it took tremendous effort to get organizations support the integrated regional approach

(I6, project manager).

This factor led to the unraveling of the multidisciplinary agreements to adhere to current financial systems.

Furthermore, as health care insurance companies tended to stick to their own contract and not follow the contract of the other health care insurers in the same region, the second strategy of PHM initiatives was to reduce ambiguity with regard to which policy, and thus which contract, was preferred in the region (all PHM initiatives). This strategy was especially important when there were two or more health care insurers with a similar or at least substantial market share in PHM regions. Regulations about contracting of insurance companies were regarded by leaders of PHM initiatives as a constraining contextual factor as insurance companies could contract care provision as they saw fit. This factor led PHM initiatives to require clarity of the non-preferred insurance company at the earliest possible stage to reduce uncertainty in financial revenues, reduce additional administrative burdens and reduce the risk of lack of support for the intervention (all PHM initiatives).

## Discussion

This study identified ten guiding principles for the organization of regional collaboration to improve pharmaceutical care in line with the TA goals: create agreement and commitment based on a long-term vision; foster cooperation and representation at board level; use the layered governance structure; create awareness at all levels; enable interpersonal links at all levels; create a learning environment; organize shared responsibility; adjust financial strategies to the market context; organize mutual gains; and align regional agreements with national policies and regulations. The strategies underlying each guiding principle interact with various contextual factors and thereby trigger different mechanisms that influence collaboration to improve pharmaceutical care. Furthermore, the guiding principles are interdependent. Therefore, it is necessary to develop and implement them together in order to realize the greatest improvements and outcomes.

Reflecting on the guiding principles, two types of guiding principles can be identified: guiding principles 1-5 predominantly represent the people management side of improving collaboration. Guiding principles 6-10 relate predominantly to the technical conditions of improving collaboration. Both types of guiding principles are interdependent as is in line with previous work (Tsasis *et al.*, 2012; D’amour *et al.*, 2008). The current study, for instance, revealed that the success of organizing shared accountability arrangements (a mainly technical guiding principle) highly depended upon getting people on board and commit to the collaborative initiative, getting people more aware of why and what changes were needed (both guiding principles that mainly address the people management side), and getting people to adopt a learning environment (a mainly technical guiding principle) that supports multidisciplinary accountability.

For guiding principles 1-5 to be effective, it is important that regional stakeholders get to know each other and gain awareness of shared regional problems in order to be able to develop a long-term regional vision. This requires contexts highlighting mutual dependence of regional stakeholders which is in line with McMillan and Chavis (1986). These authors revealed that mutual dependence is essential to create a sustainable sense of community: membership, shared emotional connection, integration and fulfillment of needs, and influence. In contrast to McMillan and Chavis, who referred to the need for leadership and power to influence the participation of stakeholders, our study indicated that building interpersonal links based on feelings of trust, openness to new perspectives, face-to-face conversations, and recognition and acknowledgment of each other’s work reinforced bidirectional influence among stakeholders. These factors are in line with the model of interpersonal relations of Lanham *et al.* (2009). Also, adding to Schein (2010), who mentioned that cooperation on the operational level is based on a feeling of security, it was shown in the present study that the same holds true on board level. The expected revenues of the shared savings contracts enhanced a sense of safety for change, which contributed to the cooperation on the board level of PHM initiatives. Creating opportunities such as expected financial revenues of shared savings contributed to multidisciplinary responsibility for improvement of pharmaceutical care and could lead to greater probability of scale-up and visibility of the PHM initiatives. Furthermore, guiding principles 1-5 resonate with the literature highlighting the importance of stepwise engagement of stakeholders in the context of reform based on a long-term vision (e.g. Breton *et al.*, 2010; Ovseiko *et al.*, 2014; Pate *et al.*, 2010). Our findings are in agreement with research on the cooperation of board members and shared leadership roles distributed across layered governance structures, which reduce disconnection between the board, management and the operational level (e.g. Buchanan *et al.*, 2007; Best *et al.*, 2012; Greenhalgh *et al.*, 2012). Lastly, our findings resonate with the literature on social connection important for creating awareness of problems and possible solutions (Scott *et al.*, 2000), and creating ways for socially reinforcing changes in working practices (Wilson, 2010).

Guiding principles 6-10 are consistent with the literature on the importance of learning environments in times when roles and working practices, professional and organizational status are in flux (e.g. Scott *et al.*, 2000; Tajfel, 1972; Fong *et al.*, 2007). Our study showed that not only problem awareness, the quality of the relationship and the amount of trust between organizations and professionals are important social forces that induce shared responsibility (Scott *et al.*, 2000; Chreim *et al.*, 2010; Wilson, 2010), but that also positive experiences within the organized learning environment helped negate resistance to change and enabled sharing multidisciplinary responsibility. Therefore, together, these elements could be regarded as a substitute for formal control mechanisms. The guiding principles also resonate with the competition literature on prices and quality (Charlesworth and Cooper, 2011). Additionally, alternative funding models have been introduced in several PHM initiatives. This study identified that the shared savings contracts have brought first experiences and goodwill within the PHM initiatives to experiment with new funding models that encourage multidisciplinary responsibility. Shared savings contracts might help modify underlying care patterns. Moreover, the relationship between the health care insurance companies and providers seems crucial for the organization of shared gains and the success of reforms in payment methods and thus the sustainability of the reform of pharmaceutical care. This is in line with previous work by Song *et al.* (2014) and Petsoulas *et al.* (2011). Furthermore, in accordance with the work of Liddy *et al.* (2013) and Kingdon (1995), changes in purchasing processes and preference policies of health care insurance companies were based on two-way exchanges between national policies and regional practice, which helped to create windows for change. Health care insurers, as key partners in the PHM initiatives, adjusted for instance national preference policies based on evidence emerging from the PHM initiatives that the new pharmaceutical formulary was the example of best practice. Lastly, PHM initiatives derived legitimacy for linking selected data to help individual patients to manage their own care, based on support expressed by a visiting political delegate. However, the lack of political acknowledgment of legal tensions related to privacy laws and regulations could hinder the progress of PHM initiatives (Kingdon, 1995), especially as experiments of combining data are technically feasible, and anticipates future constraints of data exchange as PHM initiatives engage in integrating clinical and community services.

This study adopted an evaluation method based on realist principles. This approach provides insights that other forms of synthesizing evidence would not have given (Willis *et al.*, 2016). Furthermore, the engagement of experts and knowledge users from the beginning ensured that this study is rooted in the activities of those actually undertaking collaborative efforts to achieve results in line with the TA. Moreover, ongoing engagement of experts and reference panels in understanding and refining the data renders a degree of confidence in the reliability of our analysis. However, this study has limitations. First, while our approach was systematic and rigorous, our results cannot be regarded as an exhaustive list of elements that influence the process of organizing regional collaboration to improve pharmaceutical care. On the contrary, the guiding principles identified in this study are part of an ongoing process of sharing experiences and knowledge to advance our understanding of “what strategies work in what context, and why these strategies work” to produce intended or unintended results. Second, the focus of this evaluation was on Dutch pharmaceutical care. The results of this study will probably also be applicable in fields other than those of improving pharmaceutical care as the guiding principles are based on all the components of the CAHN framework. However, more insight is needed as not all of the subcomponents as identified in the CAHN framework were addressed (Steenkamer *et al.*, 2018). The reason for this can partly be attributed to the fact that enhancing collaboration to improve pharmaceutical care only concerned the care sector while CAHN contains the (sub)components that influence collaboration across the continuum of public health, health care, social care and community services.

Improving pharmaceutical care was in most Dutch PHM initiatives the first step in a large program of change to build multiorganization and multisector collaborations designed to address both medical and social determinants of health for regional populations. Elements such as developing a multidisciplinary business plan, executing a multidisciplinary regional approach, building and improving relationships, and experimenting with new forms of funding such as shared savings contracts formed the basis for future regional collaboration to align prevention, care and welfare services. The current PHM guiding principles provide health system leaders and policy makers the necessary ingredients to choose strategies that will lead to the intended outcomes given local, regional and national constraints and opportunities. Considering the broader ambitions of PHM initiatives, future research in the guiding principles, the underlying strategies and accompanying contexts and mechanisms by which these guiding principles operate is necessary. The guiding principles are part of an ongoing process of sharing knowledge and experiences. The Dutch Monitor of Pioneer Sites Population Management will continue to monitor the Dutch PHM initiatives as they add new stakeholders and populations and seek to grow their PHM programs. In addition, we will gain insight into the learning experiences of four international PHM initiatives in 2017-2018. As a result, we will be able to enrich the current guiding principles and identify new ones, and as such will contribute to the international debate and learning with regard to the organization of collaboration across public health, health care, social care and community services.

## Conclusion

There are many factors stimulating collaboration to improve pharmaceutical care in the Dutch PHM initiatives. The ten guiding principles identified in this study will support health system leaders and policy makers to design, implement or improve strategies fostering collaboration with the aim of improving pharmaceutical care. The guiding principles must be developed and implemented together in order to realize the greatest improvements and outcomes. Health system leaders can use these principles in the contexts of their own regional setting and identify which activities and policies make most sense, given regional constrictions and chances. Improving pharmaceutical care is an incremental process. Continuous sharing of practices and understandings in implementing the guiding principles will lead to improved pharmaceutical care.

## Figures and Tables

**Figure A1:**
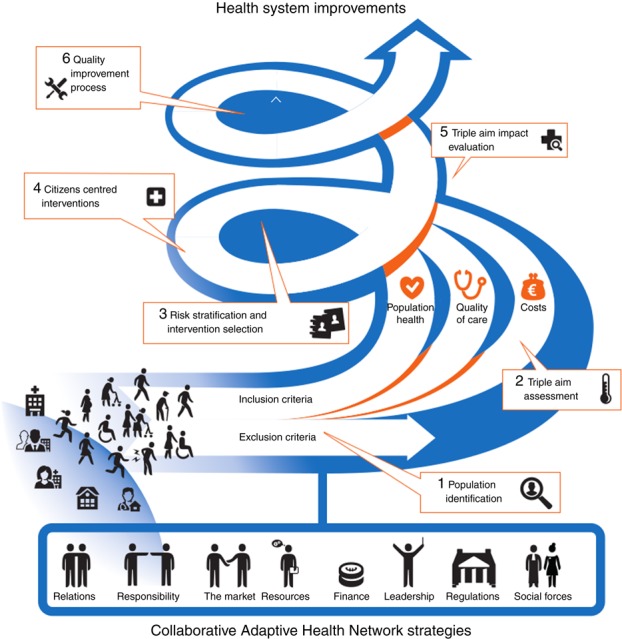
Collaborative adaptive health networks

**Table I:** Guiding principles including strategies, contexts and mechanisms underlying collaboration to improve pharmaceutical care

Guiding principles: specific strategies per guiding principle	Enabling (+) or constraining (−) contextual factors that influence the likelihood of guiding principles to be effective	Mechanisms by which these guiding principles operate
*1. Create agreement and commitment based on a long-term vision*		
a. Engage a small number of stakeholders within the care domain	+ Prior mono-disciplinary approaches with little effect on improving pharmaceutical care	Induces a sense of urgency to work together to achieve improvements on pharmaceutical care
	+ Dissatisfaction with competition among care providers in regional market	Induces readiness for multidisciplinary approach in the regional market based on a long-term vision
b. Facilitate joint development of a business plan pharmaceutical substitution	+ Increased pressure based on a growing sense of urgency to improve inefficient pharmaceutical care	Induces feelings of problem ownership
	− Internal organizational matters such as reorganizations, reallocation of capacity	Consideration of substantive, strategic and financial arguments whether to agree and commit to a small-scale project Balancing the degree of importance with regard to agreement and commitment to pharmaceutical substitution
*2. Foster cooperation and representation at board level*		
a. Create opportunities that will stimulate multidisciplinary collaboration to improve pharmaceutical care	+ Introduction of shared savings: awaiting positive results	Generates safety to show that interdisciplinary cooperation works Generates safeguarding of a financial buffer for future projects Generates motivation to put PHM initiatives “on the map”
b. Install the right people at the right time in the right place	+ or − Representation of pharmacists on the board level – or in a legal entity or steering group/project group pharmaceutical care	Representation of pharmacists on the board level or regional legal entity generates safeguarding of more involvement in other projects and in the development of pharmaceutical policy in the region Representation of pharmacists in a separate pharmaceutical steering committee/project group or legal entity generates safeguarding of more investment of time and more in-depth pharmaceutical knowledge
*3. Use the layered governance structure*		
a. Conscious use of information within the layered governance structure for escalation and facilitation purposes	+ Need to solve problems that hinder the progress of the pharmaceutical project	Generates commitment to modify behavior and working processes in line with the agreed upon protocol
b. Conscious use of skills and influencing power of PHM managers and experts within the layered governance structure for escalation and facilitation purposes	+ History of working together	Generates motivation to modify behavior without harming working relationship
	+ Differences in interests and commitment	Generates feeling of insight into differences in interest and commitment Generates confidence in launching off the pharmaceutical project
*4. Create awareness at all levels*		
a. Organize informed interaction and communication	+ Development of a toolkit pharmaceutical care – pharmaceutical formulary using data	Generates reconsideration of pharmacotherapies Generates taking into account different medical guidelines Generates adapting to a new structured way of working in accordance with the jointly developed pharmaceutical protocol
b. Stay in line with/make use of existing consultation situations between medical specialists, general practitioners, pharmacists, physician assistants and patients	+ Pre-existing quality of consultations between medical specialist, general practitioners, pharmacists, physician assistants and patients	Induces a safe situation for confrontation and awareness
c. Develop patient information and/or make it available to patients		Induces awareness Generates reduction of mistrust regarding new drug efficacy
*5. Enable interpersonal links at all levels*		
a. Join existing consultation situations between professionals	+ Increasing collaboration within primary care and between primary and secondary care	Induces trust, recognition and acknowledgment of each other’s contribution and (scientific) knowledge brought into the project to improve pharmaceutical care
b. Organize regional multidisciplinary meetings to share best practices/pharmaceutical protocol		
c. Invest in relationships between different professions		
*6. Create a learning environment*		
a. Organize adequate data input and tool development	+ or − At the start of the project, decisions made within the patient-doctor relationship were based on lack of the right information (quality, timing and level of feedback of the data)	Influences motivation of professionals to engage in the feedback loop
b. Create capacity and knowledge regarding data technology, analysis and synthesis to support the plan-do-check-act cycle	+ or − At the start of the project, insufficient capacity and knowledge regarding data technology, and analysis and synthesis of data	Induces pressure to establish either internal or external (organizations outside the population health management initiative) capacity and knowledge
*7. Organize shared responsibility*		
a. Organize new incentive design fitting regional multidisciplinary responsibility	+ Separate financial incentives did not fit the new regional agreement on multidisciplinary responsibility to improve pharmaceutical care + A growing sense of urgency to improve inefficient pharmaceutical care + Expectation that shared savings prevent shifts in responsibility − Differences between professionals and organizations regarding the design of the accountability model	Induces exploration mechanisms with regard to new incentive designs taking into account differences in cut of values and scores, setting benchmark etc
b. Organize an adherence design strongly based on social forces (peer reviewing in a multidisciplinary context)	+ Dissatisfaction with historically higher % prescription expensive drugs and mono-disciplinary responsibility +Positive experiences with the plan-do-check-act cycle	Induces a shift to multidisciplinary accountability resulting in higher market mobilization than mono-disciplinary accountability Induces a strong motivation for achieving better integrated performance
*8. Adjust financial strategies to the market context*		
a. Take into account market factors and trends regarding pharmaceutical products in the regional market	+ Market situation of pharmaceutical products differ for specific populations	Induces focus on efficiency and/or quality in order to influence price fixing
b. Organize financial insight ranging from an individual to a regional level	+ Strive for optimization of care conform accountable care principles better health, quality of care and reduction of costs growth	Induces deliberate use of financial outcomes and combining this data with clinical data and patient preferences
*9. Organize mutual gains*		
a. Focus on low-hanging fruit’ to gain quick wins	+ Discussions in society at large (among others discussions about the bonus culture of banks and the influences op the pharmaceutical industry)	Induces focus on distribution of gains
b. Put population health management initiatives “on the map”	+ Pressure within the population health management initiative to work toward a comprehensive regional program	Induces focus on achieving quick wins
*10. Align regional agreements with national policies and regulations*		
a. Indicate at the earliest possible moment where existing policies and regulations pinches implementation of new regional agreement	− The preferential policy of the health care insurers	Experiencing risk factors both at the level of the treatment relationship and at the level of the population health management initiative itself
	− Existing walls between sectors and disciplines regarding funding systems	Induces unraveling of multidisciplinary agreements to meet current funding system
b. Pursue freedom of contracting by health care insurers	− Regulations regarding contracting by health care insurers	Wish for clarity or “a go” of the non-preferred health care insurer to follow the contract of the preferred health care insurer at the earliest possible stage of the development of the regional agreements

## Appendix. The CAHN framework

The CAHN framework gives insight into how and why cross-sector collaborative strategies aiming to achieve better population health and quality of care while reducing cost growth (TA) reached specific outcomes, and the theories that contribute to these explanations. The framework contains eight components: social forces, resources, finance, relations, regulations, market, leadership and accountability (see Figure A1). Each component within the CAHN framework contains the component specific links between the strategies aiming to achieve cross-sector collaboration and the contextual factors in which these strategies are implemented, the mechanisms that explain why specific outcomes were reached, given these contextual factors, and the outcomes of strategies. Most components contain three or more subcomponents. In total, 38 subcomponents were identified. All (sub)components are based on existing theories and models that originated from scientific areas such as sociology, political science, cultural science, organizational science, economics and system dynamics. As such, the CAHN framework summarizes the available theories and insights into the relationships between strategies, contextual factors, mechanisms and outcomes of collaborative strategies cross-linking public health, health care, social care and community services.

Figure A1 represents the evaluation model of the Dutch Monitor of Pioneer sites Population Management of the Dutch National Institute for Public Health and the Environment (Struijs *et al.*, 2015; Drewes *et al.*, 2015). The figure shows the eight components that influence the process of organizing cross-sector collaboration to improve population health and quality of care while reducing the growth of health care costs (TA). In addition, the figure shows the six steps of the PHM approach necessary to implement population-centered TA interventions.

An overview of the representatives of the focus groups and interviews, the characteristics of the Dutch PHM initiatives and pharmaceutical interventions, and the interview guideline are available upon request.
